# Personal Health Information Recommender: implementing a tool for the empowerment of cancer patients

**DOI:** 10.3332/ecancer.2018.851

**Published:** 2018-07-11

**Authors:** Galatia Iatraki, Haridimos Kondylakis, Lefteris Koumakis, Maria Chatzimina, Eleni Kazantzaki, Kostas Marias, Manolis Tsiknakis

**Affiliations:** 1 Computational BioMedicine Laboratory, FORTH-ICS, Heraklion GR70013, Greece; 2 Department of Informatics Engineering, Technological Educational Institute of Crete, Heraklion GR71004, Greece

**Keywords:** medical information, recommendations, information retrieval, natural language processing

## Abstract

Nowadays, patients have a wealth of information available on the Internet. Despite the potential benefits of Internet health information seeking, several concerns have been raised about the quality of information and about the patient’s capability to evaluate medical information and to relate it to their own disease and treatment. As such, novel tools are required to effectively guide patients and provide high-quality medical information in an intelligent and personalised manner. With this aim, this paper presents the Personal Health Information Recommender (PHIR), a system to empower patients by enabling them to search in a high-quality document repository selected by experts, avoiding the information overload of the Internet. In addition, the information provided to the patients is personalised, based on individual preferences, medical conditions and other profiling information. Despite the generality of our approach, we apply the PHIR to a personal health record system constructed for cancer patients and we report on the design, the implementation and a preliminary validation of the platform. To the best of our knowledge, our platform is the only one combining natural language processing, ontologies and personal information to offer a unique user experience.

## Introduction

The explosion of the Internet and the rapid increase of available information have greatly affected the way information is accessed and distributed. More and more patients are using the Internet to look for health and medical information. As early as 2002, 80% of all adults online in the United States were estimated to have looked for health information, whereas 23% of Europeans were using the Internet to be informed about their health needs [1]. Patients, by being able to search for health information, are feeling more empowered, active and more inclined towards being involved in decision making regarding their own health [2].

However, despite the tangible benefits of this vast amount of information online, there are certain challenges as well. Besides the large volume, the quality of information is very diverse, ranging from journal articles, social media opinions, forums and so on. As such, a patient should possess the necessary skills to identify and accurately judge the relevance of information relating to his or her own case [1]. Furthermore, online information can lead to patients being misinformed, becoming distressed, and increasing the tendency towards self-diagnosis or self-treatment [3].

With this in mind, this paper focuses on current research activities related to the implementation of the Personal Health Information Recommender (PHIR). The PHIR [4] is targeted at improving the opportunities that patients have to inform themselves on the Internet about their disease and possible treatments, and providing them with personalised information and recommendations [5]. Its goal is threefold:

- To deliver relevant information to patients, based on their current profile as represented in their personal health record (PHR) data,
- To ensure the quality of the presented information by giving medical experts the chance to control the information that is given, and
- To facilitate an easy uptake of the new system, by minimising the necessary manual effort.

With these aims, our contributions are the following:

- A set of two individual apps (the PHIR search engine and the Semantic Annotator app) being able to be directly integrated into a PHR system.
- The Semantic Annotator app can be used by clinical experts to register validated, high-quality web documents and multimedia material to the platform.
- As soon as the relevant information is registered to the platform, the system indexes and semantically uplifts the available information.
- The PHIR search engine is then available to patients enabling them to search for useful health-related information. The PHIR search engine advances searching by providing an intelligent recommendation service, which exploits the individual patient profile and patient preferences to provide personalised information.

To the best of our knowledge, PHIR is the only medical information search engine and recommendation system that exploits the individual profile of the patient to provide personalised empowerment.

The whole platform has been developed in the context of the iManageCancer EU (http://imanagecancer.eu) research project for empowering cancer patients [6, 7]. The project aims to build an advanced, standards-based and scalable semantic integration environment [8], enabling seamless, secure and consistent bi-directional linking of clinical research and clinical care systems, which amongst others, will empower patients to extract the relevant data out of the overwhelmingly large amounts of heterogeneous data and treatment information. Currently, PHIR is piloted with cancer patients in Germany and Italy providing high-quality documents selected by healthcare providers.

The rest of this paper is structured as follows. The section ‘Related work’ presents related work. The section ‘Architecture’ demonstrates the PHIR architecture, describing in detail the available components. Then, the section ‘Evaluation design’ demonstrates the design of our evaluation and a preliminary validation of the platform. Finally, the section ‘Conclusion and discussion’ concludes the paper and discusses future directions.

## Related work

There are already several approaches aimed at providing patients with search engines with high-quality medical information such as WebMD (http://www.webmd.com), MayoClinic Patient Care (http://www.mayoclinic.org/patient-care-and-health-information), Medicine Plus (http://www.nlm.nih.gov/medlineplus) and so on. However, these engines provide a rather limited set of information and they are not dynamically adapted according to the patient’s preferences or medical history, which is true in our case.

One of the most well-known search engines is HONSearch [9], which aims to improve the quality of information aimed at both patients and medical professionals by facilitating quick access to the most relevant and up-to-date medical discoveries. Each indexed web document has to fulfil specific criteria in order to be included in the search engine and it uses a multi-stakeholder approach to include the relevant web documents. Although the approach seems to be promising, there have been some concerns relating to the quality [10] on the indexed websites. In a similar vein, but targeting medical experts, AskHermes [11] is an online question answering system aimed at answering specialised clinical questions.

Alternative approaches already exploit the profiles stored in personal health systems [12, 13] to automatically present useful information to patients. For example, adverse drug effects [14] are an alerting system to inform patients about potential risks and the adverse effects of the medications they receive. STEPPS [15] is another approach in the same arena, trying to personalise the automatic retrieval of health information using profiling information. The information is retrieved from an electronic patient record. However, the lack of semantics leads to poor results and the lack of a search engine to allow patient interaction limits the patient’s options.

Another work that focuses on combining social data with a search engine in order to provide valuable information about side-effects, medications and to track the geographic location of influenza epidemics is [16]. Although this approach identifies that added information can really improve the quality of the recommendations offered to the end users, the results are not personalised according to the individual profile.

Furthermore, Stratigi *et al* [17] exploit semantic information available through the International Statistical Classification of Diseases and Related Health Problems 10th Revision (ICD-10) taxonomy to provide recommendations to a small group of patients. However, they do not offer a search engine where users can look for their interested topic. In addition, in [18], semantic information is being exploited for enhancing the results of a recommendation service that facilitates the retrieval of results from electronic health records. Their results show that the quality of the results is highly increased when query recommendation using this information is used. Our approach indeed moves in this direction, combining semantics with recommendations but this time offering high-quality web documents to patients.

This approach builds upon the Interactive Empowerment Services developed in the p-Medicine project [19, 20], where a preliminary conceptualisation of the current approach was implemented. To the best of our knowledge, PHIR is the only system exploiting patients’ profiles to provide both automatic and nonautomatic high-quality information to patients employing semantics, reasoning and also exploiting user preferences.

## Architecture

**Figure 1.**
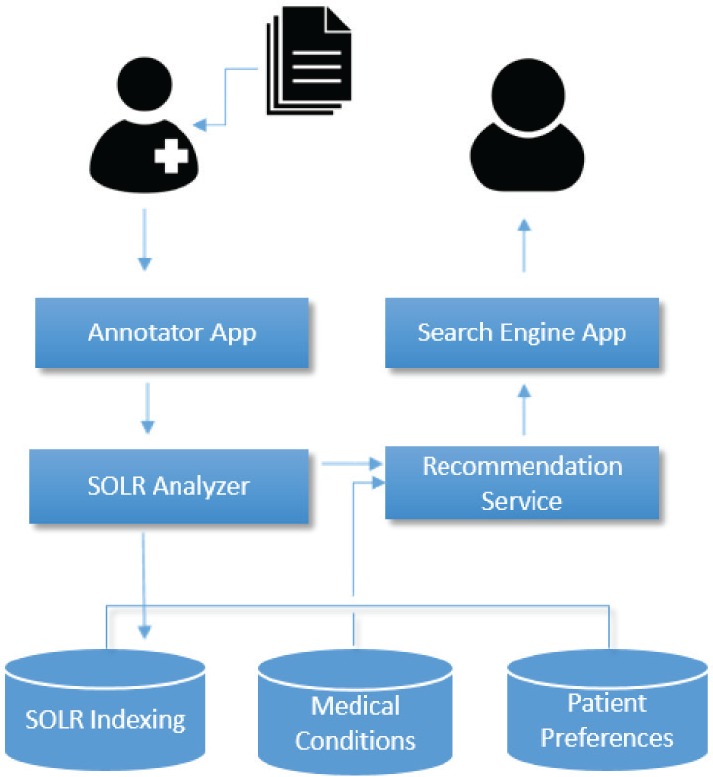
PHIR architecture: the architecture schema of the PHIR. The different modules of the system are described in this schema.

The architecture of the system is shown in Figure 1 and consists of three layers: the database layer which includes three different databases, the service layer which includes two services and the front-end layer which includes two individual apps (one for the physician and one for the patient). The PHIR has been integrated into iPHR (the PHR system developed within the iManageCancer project) as a set of individual applications. We analyse in detail each one of the aforementioned modules.

### PHIR for physicians

The first step in providing useful information to patients is the identification of useful, reliable, high-quality online health information and its appropriate and efficient use. To cope with the unprecedented volume of healthcare information available on the Internet, PHIR uses domain experts (doctors and health care providers) in order to identify and register appropriate web documents. Currently, there are indexed 855 distinct URLs selected by our domain experts for the cancer domain, also available through the web page of the engine.

#### The Annotator

**Figure 2.**
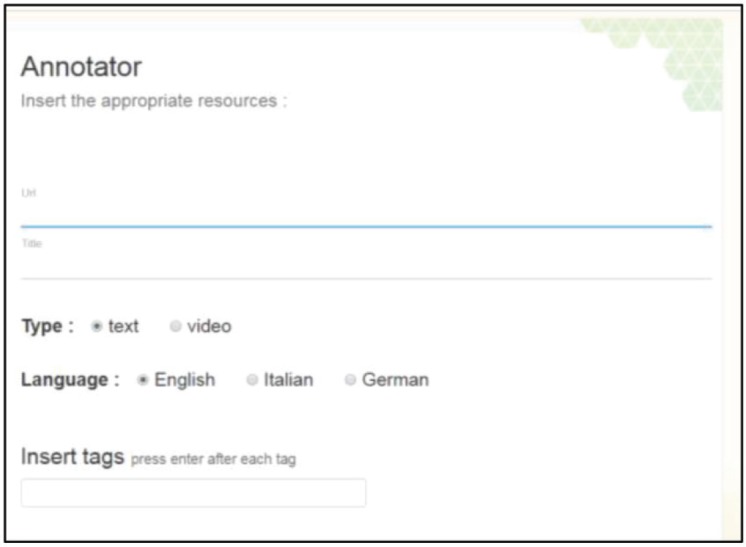
The Annotator app interface: the interface of the annotator application.

Using the Annotator app, an expert is able to register external links to documents and videos that contain useful information to be further elaborated. Those documents are high-quality web resources (web pages, pdfs, docs, online videos, etc.) selected carefully by the aforementioned domain experts, targeting patient’s needs. The interface of the app is shown in Figure 2.

In order to register an entry, domain experts just have to fill in a form using the Annotator tool including the URL of interest, a title that describes it, the type (text or video), the language of the content and tags (not obligatory). The tags and the textual content of each URL are parsed using natural language processing, as a pre-processing step, in order to identify the part of speech per word and lemmas. Lemmas of nouns are then matched against concepts from the biomedical ontology SNOMED CT using the Apache Solr server (http://lucene.apache.org/solr/), performing similarity matching. The identified terms of the SNOMED CT which have a high similarity to the available document/tags are automatically stored along with the specific web resource (document).

Besides the individual web document indexed, a web crawler systematically browses the available URLs in this web document in order to retrieve and index the contents of the entire website. An example is shown in Table 1. To achieve that, only the URLs found within the same domain name are inserted and indexed in the data server. An interesting feature of our Annotator is that it supports multiple languages, i.e. English, German and Italian and the corresponding official translations of SNOMED CT. We have to note that other ontologies can be used as well and actually using them is only a matter of providing the corresponding ontology files to the Annotator. However, we selected SNOMED-CT due to its extensive coverage of medical terms and the available translations in all three mentioned languages.

### PHIR for patients

#### The recommendation service

The results presented to the patient, by the PHIR Search Engine are provided by the recommendation service. The service considers the following databases to make the results of the query as personalised as possible:

- Patient preferences database: this database contains user preferences that are acquired as the patient browses the results presented to him/her. User’s selections and rating for a specific search result are logged to augment similar future searches.
- Medical conditions database: the medical conditions of the patient as they have been logged by the patient himself. The entries in the database are annotated using SNOMED-CT, Logical Observation Identifiers Names and Codes (LOINC) and RXTerms terms while the patients do data entry. We have to note that numerical fields are not annotated.
- SOLR indexing database: the database with the auto-generated tags from SNOMED-CT along with the annotated tags that the domain experts have provided.

The recommendation service is the bridge among the patient’s information, preferences and the indexed documents. The content that is identified as relevant is scored according to its dynamic relevance to the patient. In addition to the objective relevance, the system takes into account subjective information such as the content, which the patient has already seen, or the type of content the patient prefers (articles for the general public or specialist information, text or image, etc.).

**Figure 3.**
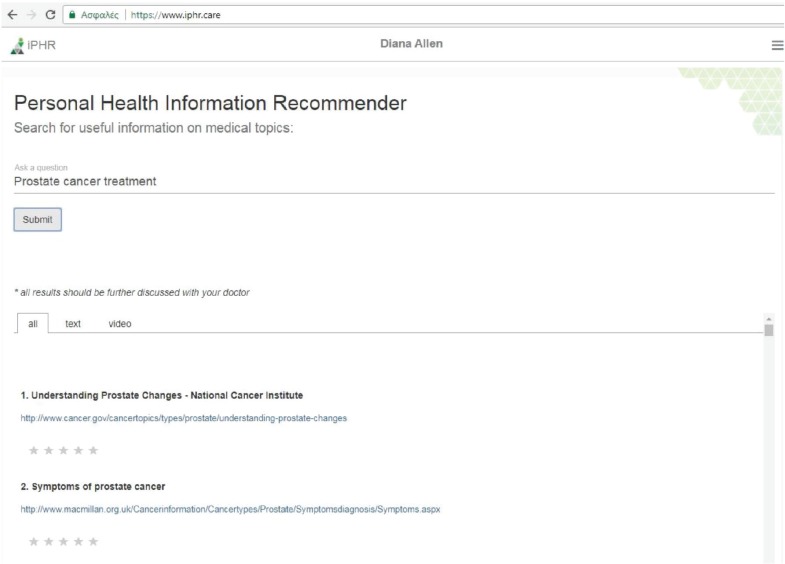
Search Engine app interface: the interface of the search engine application.

Semantic search has been proven efficient even for challenging domains such as the biomedical resource discovery [21]. The algorithm used for semantic search in the PHIR follows a gradual combination of the different results based on the user’s profile and the user’s query as shown in Figure 3.

The results are presented as a list, containing the SOLR response set to questions in the following hierarchy:

- Union of answers based on the user’s provided medical profile and the user’s given query
- Remaining answers based on the user’s provided medical profile
- Remaining answers based on the user’s query

The sorting of SOLR answers is based on SOLR analysis and indexing process.

The iPHR supplies feedback about the displayed information from the patient, either implicitly or explicitly, such that the relevance of the displayed information can be adopted over time. The process of adapting content to specific user needs can be thought of as two main sub-processes. The first sub-process involves understanding what content can be most relevant to the current user’s interests, and how this content should be organised. The second sub-process involves a decision on how to effectively present the selected content to the user.

#### The PHIR search engine application

Using the iPHR system, the patient, besides logging and reviewing their medical information, is able to search for relevant, high-quality information using an intelligent search engine provided as an individual iPHR app. The interface of the search engine is shown in Figure 3. The Search engine app sends the user query to the recommendation service and then the app visualises the returned results. The returned results are shown in tabs. A tab includes all the results and the next two tabs have them separated by type (text and video).

As well as searching for relevant results, the patient is able to rate a result according to his/her opinion using a scale of one to five. The clicks and the rankings of each user are stored in the patient preferences database, which in turn enhances the personalisation factor of the searching mechanism in future searches.

#### The PHIR search service

**Figure 4.**
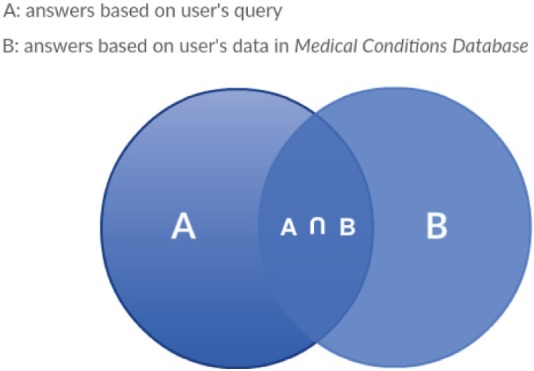
Search engine results: a Venn diagram that represents the search engine results and their combination.

**Figure 5.**
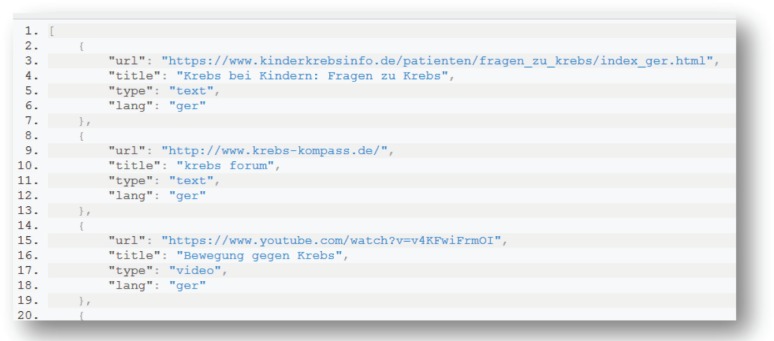
Example of data representation in SOLR: an example of a document to be inserted in SOLR and its fields. Each field has a name and content.

The PHIR Search Engine is also available to external applications through an application programming interface (API). Using the available API, an application can forward a query to the PHIR in order to receive the results in a list. An example of the JavaScript Object Notation (JSON) results returned is shown in Figure 4 whereas the corresponding JSON results returned are shown in Figure 5.

The returned results can then be visualised and processed appropriately by the external application.

## Evaluation design

In this section, we present the design of our initial evaluation in order to test the PHIR system. To evaluate our system, as already mentioned, domain experts from the iManageCancer project have already registered a plethora of web documents. Those documents contain high-quality information for patients in the cancer domain, in three languages: English, German and Italian. Those documents were selected by contact persons in the cancer domain from the (a) Information Office at the cancer charity Tenovus Cancer Care (http://www.canceractive.com/cancer-active-page-link.aspx?n=2772), (b) *e*cancer (http://ecancer.org), (c) the European Institute of Oncology (https://www.ieo.it) in Milan and (d) the University Hospital of Saarland (http://www.uniklinikum-saarland.de/en/).

For quality assurance, norms defined by the International Organization for Standardization (ISO) such as the ISO/International Electrotechnical Commission (IEC) 25000 standard (http://iso25000.com/index.php/en/iso-25000-standards) have been used as a reference model. PHIR components passed (a) a technical verification assuring that each component consistently produces the expected results, (b) a technical validation assuring the quality of the desired outcome conforming to user needs and intended uses and (c) an internal evaluation demonstrating that the system has a positive benefit to the end users. Table 2 provides an overview of the validation and evaluation results for each sub-characteristic proposed by the ISO/IEC 25000 standard. Performance, resource utilisation and capacity were measured using a web performance tool (https://www.webperformance.com/), while the rest of the characteristics and sub-characteristics have been measured based on the functionality of the system and user requirements.

Furthermore, physicians participating in the iManageCancer consortium from the European Institute of Oncol-ogy and the University Hospital of Saarland guided the developers and performed an initial evaluation. They provided feedback with the main objective to create a tool that they would recommend to their patients. After two internal iterations (evaluation workshops with physicians as end users), and a preliminary evaluation using a small set of patients at a cancer conference in Milan, the PHIR reached its stable stage (version 1.0 of the tool). The results of the internal evaluation indicate the potential for the tool to help patients to gather precise information about their diagnosis and treatment.

The main evaluation of PHIR will be based on the pilots of iManageCancer, which will assess the benefit of the PHIR and the PHR platform as a whole. The iManageCancer platform will be evaluated in two healthcare domains for children and adults. The enrolment of the patients has already started and is estimated that by mid-2018, 60 nephroblastoma patients and their parents will participate in the children’s pilot and 120 prostate and breast cancer patients will participate in the adult’s pilot. Both the pilots will have a duration of at least 6 months. Patients will answer usability questionnaires during the period of the pilot. The system also logs the time each patient spends using PHIR and how frequently he/she has used the system. The personalised preferences (user can rate if the proposed content is close to what he/she expected to view) are also logged. The results of these three different sources will identify the usability and the usefulness of our search engine, whereas analytics will be used to provide further insights on the PHIR usage [22]. Although the number of patients is limited, we expect this to give us some directions and hints for the future development of our engine.

## Conclusion and discussion

The changing nature of information distribution due to the evolution of the Internet has important implications for health care. Given the wide use of the Internet in providing medical information, providing patients with appropriate content might further enhance the patient’s education and experience. The validity and the quality of the available healthcare information on the Internet is an area of major concern mainly because these have not been well-documented [23]. Although healthcare professionals should continue to strive to be the main source of information for patients, we should also be aware that most will continue to use the Internet to gather information [24]. Berg *et al* [25] concluded that the optimal solution for patients is to be guided by healthcare providers to more optimal resources over the web. Delivering accurate sources to the patient increases his/her knowledge and changes the way of thinking which is usually referred to as patient empowerment. As a result, the patient’s dependency for information from the doctor is reduced. Moreover, patients feel autonomous and more confident about the management of their disease [26].

The PHIR platform focuses on making the available information timelier and more relevant with respect to dynamic influences on the individual patient’s treatment. While PHIR focuses on patient’s recommendations, the architecture of the system can be applied to other fields, such as tools discovery [21], by replacing the ontologies and the search cohort. The idea behind PHIR is that even if two patients suffer from the same disease and they are in the same phase of treatment, their interests regarding the available information may differ based on various factors. For example, patients might have different medical backgrounds which are not directly related to the treatment, they might receive additional drugs due to other, independent treatments, or they might be affected by other external factors such as the weather in case of allergies. More subjective factors include patient preferences for more simple or more complex information. To the best of our knowledge, no other system providing medical information is able to be dynamically adapted in such a diverse environment. After the end of the pilots, we plan to provide the PHIR source code for free under Apache License 2.0.

## Conflicts of interest

The authors declare no conflict of interest.

## Authors’ contributions

Galatia Iatraki and Maria Chatzimina implemented the tool and wrote the implementation part of the paper. Haridimos Kondylakis and Lefteris Koumakis designed the algorithms behind the implementation and described the corresponding parts of the paper. Kostas Marias and Manolis Tsiknakis supervised the development and contributed with the introduction and the discussion sections.

## Figures and Tables

**Table 1.** Crawler extracted information—an example: the table illustrates the information extracted from the crawler mechanism which is a part of the Annotator app. The first column shows an example of a URL imported in the Annotator by an expert. The second column shows the URLs extracted from the given one that will also be imported in the system.

URL: Imported by the expert	URLs: Imported by the web crawler mechanism
http://www.cancerresearchuk.org	http://www.cancerresearchuk.org/about-cancer/ http://www.cancerresearchuk.org/about-cancer/type/breast-cancer/ http://www.cancerresearchuk.org/about-cancer/type/lung-cancer/ http://www.cancerresearchuk.org/about-cancer/type/ http://www.cancerresearchuk.org/about-cancer/cancers-in-general/

**Table 2.** Evaluation results.

Measurement	Description	Result
Functional suitability		
Functional completeness	Degree to which the set of functions covers all the specified tasks and user objectives.	Seven requirements/seven completed
Functional correctness	Degree to which a product or system provides the correct results with the needed degree of precision.	True
Functional appropriateness	Degree to which the functions facilitate the accomplishment of specified tasks and objectives.	True
Compatibility		
Coexistence	Degree to which a product can perform its required functions efficiently while sharing a common environment and resources with other products	True
Interoperability	Degree to which two or more systems, products or components can exchange information and use the information that has been exchanged.	True
Security		
Confidentiality	Degree to which a product or system ensures that data are accessible only to those authorised to have access	True
Authenticity	Degree to which the identity of a subject or resource can be proved to be the one claimed.	True
Performance efficiency		
Time behaviour	Degree to which the response and processing times and throughput rates of a product or system, when performing its functions, meet requirements.	Average response time: 1,310.57 ms
Resource utilisation	Degree to which the amounts and types of resources used by a product or system, when performing its functions, meet requirements.	Min response time: 187 ms, max response time :12,024 ms
Capacity	Degree to which the maximum limits of a product or system parameter meet requirements.	Network I/O: 40.24 KB/s; memory: 13.82%; CPU: 5.72%; connections: 13
